# Personality predicts song complexity in superb fairy-wrens

**DOI:** 10.1098/rsos.241497

**Published:** 2025-04-16

**Authors:** Diane Colombelli-Négrel, Andrew C. Katsis, Lauren K. Common, Sonia Kleindorfer

**Affiliations:** ^1^ College of Science and Engineering, Flinders University, Adelaide, South Australia, Australia; ^2^ Konrad Lorenz Research Center for Behavior and Cognition, University of Vienna, Grünau im Almtal, Austria; ^3^ Department of Behavioral and Cognitive Biology, University of Vienna, Vienna, Austria

**Keywords:** aggressiveness, exploration, learnt signals, Maluridae, sexual signalling

## Abstract

In birds, singing behaviours play a critical role in mating and territory defence. Although birdsong can signal individual quality and personality, very few studies have explored the relationship between individual personality and song characteristics, and none has investigated this in females. Here, we examined the relationships between song complexity and two ecologically relevant personality traits (exploration and aggressiveness) in wild superb fairy-wrens (*Malurus cyaneus*), a species in which both sexes learn to produce complex songs. First, we assessed personality in males and females (including juveniles) by quantifying their exploration behaviour (novel environment test) and aggressiveness (mirror stimulation test) during short-term captivity. After birds were released, we recorded their songs over several months to assess individual variation in song complexity (i.e. element types per song and syllables per song) in relation to personality. Regardless of their sex or life stage, individuals that were more exploratory had more element types per song. Additionally, more aggressive individuals produced songs with fewer syllables, and more aggressive fledglings, but not adults, had more element types per song. Our study supports the idea that both male and female birds can advertise their personality when singing, which may be important for mate choice.

## 1. Introduction

In wild animals, consistent individual differences in behaviour—also termed personalities—can have important consequences for both individual fitness [1–3] and ecological processes, as individuals with different personalities respond differently to environmental change and can vary in their invasion or dispersal ability, as well as their risk of extinction [4–7]. Personality traits have most commonly been measured along one of five behavioural axes, namely boldness, exploration, activity, aggressiveness and sociability [8–11]. Animal personality may influence and interact with sexual signalling in many ways, by affecting behavioural attributes desirable to mates, energy expenditure or how individuals engage with their environment [12,13]. Yet, the proximate and ultimate interactions between personality and signalling remain largely unexplored.

Individuals with different personality types are expected to engage in different strategies to manage the trade-off between survival and reproduction: bolder/more aggressive individuals tend to have higher reproductive success but lower survival, while shyer/less aggressive individuals tend to be more risk averse [14–17], but see [18]. Individuals should, therefore, advertise behavioural attributes that would help their offspring succeed, which may, in turn, influence mates’ preference for certain personality types [19,20]. Individuals could then select mates with personality phenotypes that align with (i.e. assortative mating) or are in opposition to their own personality (i.e. disassortative mating) [12], which may provide a fitness advantage. For example, in great tits (*Parus major*), pairs with opposing exploration phenotypes produced fledglings in the best condition [21].

If sexual signalling and personality are, indeed, linked within individuals, there may be energetic or risk-associated trade-offs between these traits [22,23]. Sexual signals, such as songs or bright plumage, can be energetically costly to produce [24,25], which may lead more exploratory individuals to prioritize short-range and less expensive signals over longer range signals, as their personality phenotype should naturally increase encounter rates with mates [13]. Similarly, some personality phenotypes may be costly in some contexts. Boldness and exploration, for example, can increase predation risk [1], while aggressiveness during agonistic interactions may increase injury risk [26,27]. As a result, bold and exploratory individuals might need to reduce any signalling that makes them conspicuous to predators [28] and instead produce signals that are more effective at close range, while shy or less exploratory individuals may spend less time in the open than their bold/more exploratory counterparts to lower the odds of being detected [13].

Personality may also affect how individuals interact with their environment, which could, in turn, influence their signal efficacy and learning [13,29,30]. For instance, shyer individuals may spend more time in shelters or safer habitats, while exploratory individuals may utilize larger home ranges or investigate a wider range of habitats [31,32] and thereby communicate with a larger number of conspecifics. In birds, personality could also influence the learning of sexual signals, such as song. If personality influences the exploration of different habitats [32], then more exploratory individuals might encounter a wider range of vocal stimuli and incorporate more elements into their repertoire than less exploratory individuals [33,34]. Personality also affects attentiveness to and use of social cues [35,36], as well as learning during vocal discrimination tasks [29,30]. As such, slow explorers may be better learners (and produce songs with higher complexity) because they take more time to explore their environments and assimilate more information about their environment [29,30].

Despite the limited research on how animal personalities influence sexual signalling, some studies have demonstrated that birds can advertise their personality while singing. For example, bolder and more exploratory male collared flycatchers (*Ficedula albicollis*) sang at lower posts than their shyer/less exploratory counterparts, which provided them with fitness benefits, as they paired more quickly [34]. Similarly, fast-exploring male great tits sang more frequently during key moments of female reproductive investment, such as the pre-breeding, egg laying and incubation periods [37,38]. During territory intrusions, slow-exploring great tits produced songs with shorter duration and slower element rates than more exploratory males [39]. However, none of these studies found an association between personality and any learned song characteristics, such as song complexity or repertoire size [34,37]. In addition, no study has investigated the relationship between personality and song characteristics in species where both sexes sing.

Here, we examined the relationships between song complexity and personality (exploration and aggressiveness) in wild male and female superb fairy-wrens (*Malurus cyaneus*). In this species, exploration (*R* = 0.37−0.43) and aggressiveness (*R* = 0.09−0.29) behaviours are each individually repeatable and are considered personality traits [15,40]. Both traits, measured in short-term captivity, are also reliable indicators of risk-taking in the wild (which is linked to individual differences in fitness, such as reproduction and survival) [14–18]: fast and slow explorers (compared with intermediate explorers) defended their territory more vigorously during simulated conspecific intrusions [41], and more aggressive individuals responded more strongly to predator playback than less aggressive individuals [42]. Further, fast-exploring individuals were less likely to be present in the population 12 months later, which may indicate higher mortality consistent with greater risk-taking [15]. Since personality in superb fairy-wrens seems to be established during the first year of life [40], during which time young fairy-wrens learn their songs from both parents and unrelated tutors [43], this species offers a useful system to investigate the potential relationship between personality and song learning. Superb fairy-wrens are cooperative breeders, with one dominant breeding pair often assisted by a mix of related (sons and daughters) and unrelated helpers [41,44]. While fairy-wrens are highly territorial during the breeding season, during the non-breeding season they often combine with neighbouring groups to form larger foraging flocks ([45]; Colombelli-Négrel D.,2019-2025 pers. obs.), thereby increasing their exposure to different vocal stimuli. Based on this, we expected that the most likely mechanism by which personality affects song learning is by influencing the number of conspecifics an individual interacts with and learns from. Therefore, we predicted that more exploratory individuals would have higher song complexity (larger repertoire, more element types and rare elements) than less exploratory individuals, as they are expected to investigate more habitats and encounter more conspecifics from which to learn their songs. Bolder and more aggressive birds tend to be more routine-like and less innovative than their shyer/less aggressive counterparts [46,47]. Therefore, we predicted that more aggressive birds would have lower song complexity compared with their less aggressive counterparts.

## 2. Material and methods

### 2.1. Study site and species

We obtained personality scores and song recordings between August 2019 and December 2023 at Cleland Wildlife Park (34°58′S, 138°41′E), South Australia. Superb fairy-wren territories at this site have been monitored continuously since 2008, and all birds are colour-banded (e.g. [43,48]). Superb fairy-wrens are insectivorous passerines that occur in southeastern Australia [49]. Breeding generally begins between August and October and ends around February of the following year [43,50,51]. Fledglings remain in their natal group for several months after leaving the nest [49]. Adults are sexually dichromatic and dimorphic: females are brown with red legs, bills and lores, and males are either brown (in non-breeding plumage) or black and blue (in breeding plumage) with black legs and bills [49]. Juveniles (<1 year old) are visually identical to females until 1 year old and thus were sexed using molecular techniques, as described in [52]. Individuals that are categorized as juveniles in this study were only assayed and recorded as juveniles (i.e. within the first four months of their life). For individuals that were categorized as adults, we only included data collected after they reached 1 year old.

**Figure 1.**
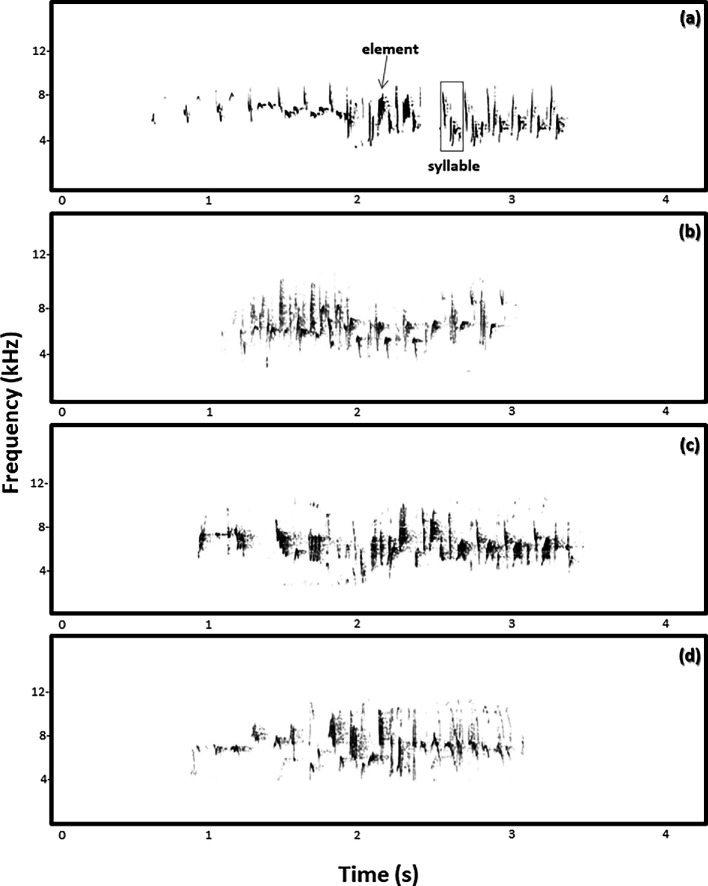
Examples of four different superb fairy-wren chatter songs from (a) an adult male, (b) an adult female, (c) a male fledgling and (d) a female fledgling. We considered an element as a single trace on the spectrogram and a syllable as a unit of elements separated by less than 1 s (examples of each indicated in figure 1a).

Fairy-wrens produce three different song types: (i) a complex type I song (referred to as ‘chatter song’) [43] (ii) a trilling type II song [53] and (iii) a type III alarm song [54]. In this study, we focused on the chatter song, as it is used for mate choice and territory defence and is sung by both sexes [41,55,56]. Chatter songs typically contain approximately 50 elements and approximately 10 element types per song ([43]; see also figure 1). In superb fairy-wrens, male and female songs can differ in frequency, duration, complexity and usage of certain element types [43,51,57]. Juveniles start singing subsongs approximately 16 days post-fledging and full songs approximately 50 days post-fledging [43]. The sensory period for vocal learning in superb fairy-wrens begins in the egg [50,51,58]. There is no available information regarding when they stop learning their songs; however, recordings of the same individuals over multiple years showed that they continue to include new elements into their songs as adults (Colombelli-Négrel, unpublished data 2019-2025) and previous study on the same population demonstrated that both males and females retain plasticity in their adult song [59], suggesting that they might be open-ended learners.

### 2.2. Exploration scores

We assessed exploration behaviour during short-term captivity using a novel environment test following procedures previously described in [40]. Once subjects were captured using mist-nets, we took morphometric measurements and then immediately transferred them in cotton bags to an onsite building, where they were placed individually in a small wooden release box (170 mm × 115 mm × 90 mm) and allowed to acclimate for 5 min. We then raised the door of the release box to allow the bird to enter the novel environment: a metal flight cage (700 mm × 450 mm × 450 mm) with 3 wooden perches and divided into 13 sectors that the bird could potentially visit (the three perches, four floor quadrants, four cage walls, the cage ceiling and the release box). We had previously covered all sides of the cage, except one, with an opaque fabric that visually isolated the bird from its surroundings, and placed a small video camera (GoPro Hero 7 Black, GoPro, Inc., USA) on the open side to record the bird’s movements within the cage. We scored the video recordings using the software Solomon Coder (v. beta 19.08.02) and, as our measure of exploration, quantified each bird’s *‘total sector visits’* (number of cage sectors visited in the first 5 min following emergence, including repeat visits to the same sector). The bird’s colour bands were visible on the videos, and thus scoring was not conducted blind to the bird’s identity; however, the scorer was blind to each bird’s song characteristics. As exploration is a demonstrated personality trait in superb fairy-wrens (i.e. responses to the novel environment are repeatable across trials) (*R* = 0.37−0.43; [15,40]), and individuals were measured more than once in this study (mean ± s.e. trials per individual = 1.54 ± 0.71; range 1−4), we used their mean exploration score for our analyses. Fairy-wrens show no evidence of habituation towards the novel environment over time, with no change in exploration score across repeated trials [40].

### 2.3. Aggressiveness scores

After the 5 min novel environment test, we immediately started a mirror stimulation test. To do so, we remotely raised a curtain located at one end of the cage to reveal a mirror (300 mm × 400 mm) and scored the total time (in secs) that the bird spent in the three sectors closest to the mirror over a 3 min period (*‘time close to mirror’*). As aggressiveness is also a demonstrated personality trait in superb fairy-wrens (i.e. responses to the mirror test have been shown to be repeatable across trials) (*R* = 0.09−0.29; [15,40]) and individuals were measured more than once in this study (mean ± s.e. trials per individual = 1.54 ± 0.71; range 1−4), we used their mean aggressiveness scores for our analyses. All birds were recaptured by hand after the two behavioural tests and immediately released within their territory. Birds were never tested more than once per day. When birds were tested over multiple trials, we recaptured them each time within their natal territory as described above. The mean ± s.e. interval between the first and last trial was 39 ± 4 days (range 20−59) for fledglings and 241 ± 23 days (range 32−428) for adults [40].

### 2.4. Song recordings and analyses

We recorded all individuals opportunistically throughout the breeding season in the same year of their capture for personality testing. For all individuals, we attempted to record as many songs as possible by visiting all territories 1−2 times per week. Individuals were identified visually using their colour bands and recording started as soon as a target bird was identified. We recorded all singing birds within a territory during a recording session. To ensure that each song could later be attributed to the correct bird, we narrated on the recording the bird’s colour bands immediately after it sang. We continued our recordings until singing stopped or for a maximum time of 20 min (whichever was longer). This resulted in recordings that varied in length. We only recorded songs when the weather conditions were optimal (i.e. no or low wind and no rain). Cleland Wildlife Park is a highly visited tourist destination and so birds at the study site were habituated to human presence. However, recordings stopped if disturbance from other birds occurred. We only recorded juveniles from 50 to 120 days post-fledging to ensure that we captured crystallized songs and that all juveniles were captured at a similar stage of song development. Adults were only recorded once they were 1 year old or older. We recorded all songs (from approx. 5 m) as broadcast wave files (16-bit, 24 kHz) with a directional microphone (Sennheiser ME67; Sennheiser electronic GmbH & Co., USA) connected to an audio recorder (Zoom H6 recorder; Zoom North America, USA).

We analysed all recordings with Raven Pro 1.6 (Cornell Lab of Ornithology Bioacoustics Research Program, Ithaca, New York) using the Hann algorithm (Discrete Fourier Transform (DFT) = 512 samples, frequency resolution = 124 Hz, time resolution = 11.6 ms and frame overlap = 50%). From the spectrograms, we categorized element types visually for each song according to a previously published element library for our study population (element types were as follow: A, F, Fl, G, K, L, O, P, Q, R, T, U, V, W, Z and Zn; [43]). We considered an element as a single trace on the spectrogram and a syllable as a unit of elements separated by less than 1 s figure 1 [60]. Element types found in fewer than 5% of all the songs recorded in the population (Fl, G, K, L, P, Q, R, T, V and Zn) were considered rare [59]. Namely, element types Fl, K and P were the rarest (found in 0.5, 0.5 and 0.4% of all songs, respectively), while element types A, O and F were the most common (found in 22, 20 and 16% of songs, respectively) [59]. The rare element types did not change during the study period (electronic supplementary material, table S1). Element types for this study were scored by two independent observers. To ensure that classification of element types did not differ between the observers, we used 60 songs scored by both observers and determined an agreement score of 98.7%.

For each individual, we measured: (i) *‘repertoire’* (total number of element types produced by an individual, also referred to as ‘vocal complexity’ [43]), (ii) *‘rare element types’* (total number of element types produced by an individual that are found in fewer than 5% of all the songs recorded in the population [59]), (iii) ‘*elements per song*’ (mean number of elements per song), (iv) ‘*element types per song*’ (mean number of element types per song), (v) ‘*syllables per song*’ (mean number of syllables per song) and (vi) ‘*syllable categories per song*’ (mean number of syllable categories per song).

To estimate whether we captured the full repertoire, we plotted the number of unique element types found against the total number of songs recorded using 43 adults (14 females and 29 males) for which we recorded at least 10 songs (see also [61]). Electronic supplementary material, figure S1 shows that the full repertoire was not recorded; hence, we refer in this study to the ‘observed repertoire’.

### 2.5. Statistical analyses

We used R v. 4.3.1 [62] for all statistical analyses. Data are shown as mean ± s.e. Similar to previous studies in superb fairy-wrens [15,40], males and females did not differ in personality traits in our study (*t*‐test exploration: *t*_100_ = −1.37, *p* = 0.173; aggressiveness: *t*_100_ = 1.84, *p* = 0.069). However, we included sex as an interaction effect in our analyses to investigate whether the relationship between personality and song characteristics differs between males and females. In addition, male and female superb fairy-wrens can differ in their song characteristics and the use of certain element types [43,51,57]. As our previous work demonstrated that personality traits in this species are consistent within but not across life stages [40], we assessed whether the relationship between personality and song characteristics varies with life stage by incorporating life stage as an interaction effect in our analyses. Because we expected some collinearity between life stage and personality [40], we additionally controlled for the effect of life stage on personality traits. We did this by conducting two linear regressions, each with one of the personality traits (exploration or aggressiveness) as the response variable and life stage as an explanatory variable, and extracting the residuals from each regression. This correction allowed us to estimate the independent effect of personality and life stage on song traits.

Spearman’s correlation tests showed that recording effort (number of songs recorded per individual) strongly correlated with some of our song characteristics (table 1). Although more exploratory birds did not sing more than less exploratory ones (electronic supplementary material, figure S2), we included the number of recorded songs per individual in our analyses to assess the effect of recording effort on song characteristics. As stated previously, juveniles were only tested and recorded within the first four months of their life, and adults were only tested and recorded after they reached 1 year old. Hence, individuals were only included once in our analyses, either as juvenile or as adult, but never at both stages.

**Table 1.** Results from Spearman’s correlation tests between the number of songs recorded and the different song characteristics measured in this study: (i) ‘repertoire’, (ii) ‘rare element types’, (iii) ‘elements per song’, (iv) ‘element types per song’, (v) ‘syllables per song’ and (vi) ‘syllable categories per song’. Statistically significant (≤0.05) values are marked in bold.

	repertoire	rare elements	elements per song	element types per song	syllables per song	syllable categories per song	number of songs recorded
rare elements	***ρ* = 0.95,** ***p* < 0.001**						
elements per song	*ρ* = 0.18, *p* = 0.075	*ρ* = 0.19, *p* = 0.054					
element types per song	***ρ* = 0.78,** ***p* < 0.001**	***ρ* = 0.75,** ***p* < 0. 001**	***ρ* = 0.23,** ***p* = 0.019**				
syllables per song	*ρ* = −0.08, *p* = 0.425	*ρ* = −0.08, *p* = 0. 433	***ρ* = 0.74,** ***p* < 0.001**	*ρ* = −0.18, *p* = 0.239			
syllable categories per song	*ρ* = 0.12, *p* = 0.240	*ρ* = 0.11, *p* = 0.280	***ρ* = 0.68,** ***p* < 0.001**	*ρ* = 0.18, *p* = 0.075	***ρ* = 0.81,** ***p* < 0.001**		
number of songs recorded	***ρ* = 0.507** ***p* < 0.001**	***ρ* = 0.543** ***p* < 0.001**	*ρ* = 0.156 *p* = 0.118	***ρ* = 0.336** ***p* < 0.001**	*ρ* = 0.005 *p* = 0.963	*ρ* = 0.012 *p* = 0.904	

Spearman’s correlation tests showed that some of our song characteristics were highly correlated (table 1), so we only included ‘element types per song’ (Model 1) and ‘syllables per song’ (Model 2) as response variables in our main analyses. We selected these two song variables as they would provide the most comprehensive information about individual song complexity while minimizing the number of analyses. Specifically, we anticipated that ‘element types per song’ would be most influenced by personality traits, based on our predictions. This variable was, in turn, strongly correlated with repertoire and number of rare elements, while syllables per song was strongly correlated with elements per song and syllable categories per song (table 1). For transparency, we present the results for the remaining song characteristics in electronic supplementary material, tables S2 and S3.

We used the ‘lmer’ function in the *lme4* package v. 1.1−32 [63] to analyse song characteristics using linear mixed models. Each model included a different song variable as the response variable. All models initially included four interaction terms [the residuals for ‘*total sector visits*’ (our measure of exploration corrected by life stage) × sex (male, female), the residuals for ‘*time close to mirror*’ (our measure of aggressiveness corrected by life stage) × sex, the residuals for ‘*total sector visits*’ × life stage (adult, fledgling) and the residuals for ‘*time close to mirror*’ × life stage], as well as the number of songs recorded per individual (recording effort), as fixed effects, and territory ID as a random effect (to account for the inclusion of multiple individuals per territory). Models were subsequently simplified by backwards stepwise removal of non-significant (*p* > 0.05) interaction terms. In our results, we present outputs from both the full and reduced models. All model diagnostics and visualizations were performed using the packages *DHARMa* v. 0.4.6 [64], *car* v. 4.3.3 [65] and *ggplot2* v. 4.3.2 [66]. Variance Inflation Factor (VIF) values were ≤3, confirming no issue with collinearity between predictor variables.

## 3. Results

We recorded 1034 chatter songs (6–26 songs per individual, mean ± s.e. = 10.14 ± 0.39 songs) from 102 individuals from which we also obtained personality data (40 females and 62 males; 76 adults and 26 juveniles). Exploration behaviour (*‘total sector visits’*) ranged from 2 to 340 sector visits (mean ± s.e. = 105.43 ± 6.82). Aggressiveness (‘*time close to mirror’*) ranged from 0 to 160 s spent near the mirror (mean ± s.e. = 45.14 ± 4.47). Observed repertoire size was between 6 and 16 elements (mean ± s.e. = 11.66 ± 0.23). The number of rare element types per song varied between 1 and 10 (mean ± s.e. = 5.91 ± 0.22). Table 2 summarizes repertoire size, number of rare element types, number of elements per song, number of element types per song, number of syllables per song, and number of syllable categories per song by sex and life stage.

**Table 2.** Summary of the different song characteristics (mean ± s.e.) measured in this study (*n* = 102 individuals).

song characteristics	males (*n* = 62)	females (*n* = 40)	adults (*n* = 76)	juveniles (*n* = 26)
repertoire	11.69 ± 0.32	11.73 ± 0.34	11.80 ± 0.28	11.27 ± 0.45
number of rare element types	6.02 ± 0.29	5.85 ± 0.33	6.08 ± 0.25	5.42 ± 0.41
number of elements per song	33.94 ± 0.83	35.10 ± 1.30	36.09 ± 0.82	29.60 ± 1.02
number of element types per song	6.21 ± 0.15	6.29 ± 0.17	6.20 ± 0.12	6.32 ± 0.25
number of syllables per song	6.24 ± 0.28	6.78 ± 0.50	7.10 ± 0.27	4.58 ± 0.46
number of syllable categories per song	1.56 ± 0.07	1.61 ± 0.10	1.69 ± 0.06	1.24 ± 0.11

**Figure 2.**
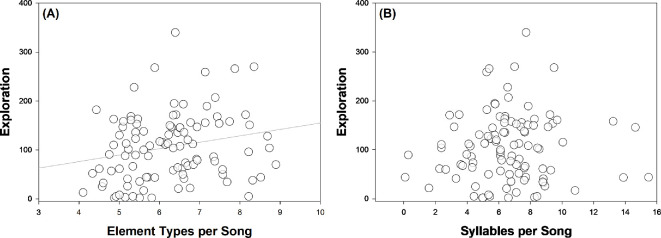
Relationships between exploration (‘*total sector visits’* in a novel environment test) and song characteristics in superb fairy-wrens (*n* = 102 individuals). Data are presented for (A) ‘*element types per song*’ (mean number of element types per song) and (B) ‘*syllables per song*’ (mean number of syllables per song).

**Figure 3.**
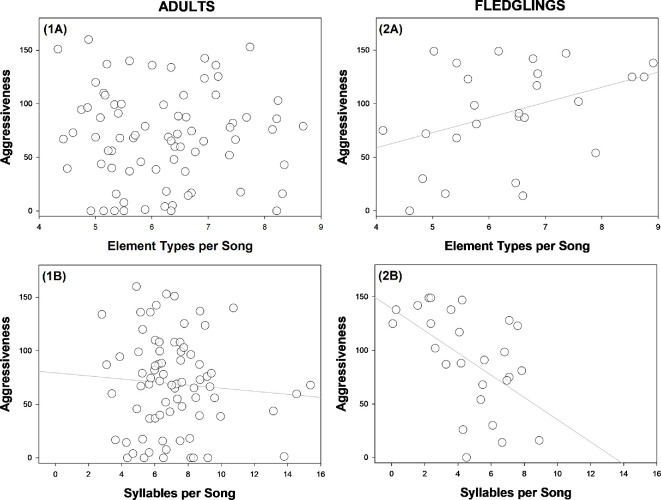
Relationships between aggressiveness (‘*time close to mirror*’ in a mirror stimulation test) and song characteristics in superb fairy-wrens, grouped by life stage (*n* = 102 individuals). Data are presented for (A) ‘*element types per song*’ (mean number of element types per song) and (B) ‘*syllables per song*’ (mean number of syllables per song). Data are presented separately for (i) adults and (ii) fledglings.

Individuals that were more exploratory had more element types per song (table 3; figure 2). There was a significant interaction effect between life stage and aggressiveness: more aggressive birds had more element types per song, but this pattern was only present in fledglings (table 3; figure 3). The number of element types was not predicted by sex (nor by the interaction between personality traits and sex) (table 3). The number of element types per song increased with the number of songs recorded per individual (table 3). The random effect ‘Territory ID’ significantly accounted for variance within element types per song (variance = 0.24, Likelihood Ratio Test (LRT) = 6.00, d.f. = 1 and *p* = 0.014).

**Table 3.** Output from linear mixed models testing the relationships between personality and song characteristics (element types per song or syllables per song) in superb fairy-wrens (*n* = 102). Full models included exploration (‘*total sector visits’* in a novel environment test; residuals), aggressiveness (‘*time close to mirror’*; residuals), sex (male, female), life stage (adult, fledgling), number of songs recorded, and four interaction terms as fixed effects, and territory ID as a random effect. Statistically significant (≤0.05) values are marked in bold. Models were simplified by backwards stepwise removal of non-significant interaction terms. The table presents full models as well as reduced models.

fixed effects	estimate	s.e.	*t*	*p*
*model 1 (element types per song)—full model*				
*intercept*	5.40	0.34	15.83	**<0.001**
exploration	0.33	0.18	1.84	0.069
aggressiveness	−0.14	0.20	−0.68	0.498
sex [male]	−0.18	0.20	−0.88	0.379
life stage [fledgling]	0.21	0.24	0.87	0.384
exploration × sex [male]	−0.18	0.22	−0.78	0.435
aggressiveness × sex [male]	0.04	0.23	0.20	0.845
exploration × life stage [fledgling]	0.15	0.29	0.50	0.620
aggressiveness × life stage [fledgling]	0.55	0.23	2.35	**0.021**
number of songs recorded	0.09	0.03	3.46	**0.001**
*model 1 (element types per song)—reduced model*				
*intercept*	5.44	0.33	16.23	**<0.001**
exploration	0.26	0.10	2.58	**0.011**
aggressiveness	−0.13	0.11	−1.15	0.252
sex [male]	−0.18	0.20	−0.89	0.375
life stage [fledgling]	0.20	0.23	0.87	0.385
aggressiveness × life stage [fledgling]	0.55	0.22	2.44	**0.017**
number of songs recorded	0.08	0.02	3.35	**0.001**
*model 2 (syllables per song)—full model*				
*intercept*	8.98	0.74	12.11	**<0.001**
exploration	−0.28	0.39	−0.71	0.478
aggressiveness	−0.48	0.44	−1.10	0.273
sex [male]	−1.05	0.43	−2.42	**0.018**
life stage [fledgling]	−3.20	0.51	−6.26	**<0.001**
exploration × sex [male]	0.27	0.48	0.56	0.579
aggressiveness × sex [male]	0.27	0.49	0.55	0.586
exploration × life stage [fledgling]	0.65	0.63	1.02	0.308
aggressiveness × life stage [fledgling]	−0.80	0.50	−1.60	0.114
number of songs recorded	−0.11	0.05	−2.01	**0.048**
*model 2 (syllables per song) – reduced model*				
*intercept*	8.96	0.74	12.14	**<0.001**
exploration	0.02	0.22	0.09	0.926
aggressiveness	−0.47	0.22	−2.14	**0.035**
sex [male]	−1.10	0.43	−2.57	**0.012**
life stage [fledgling]	−3.16	0.51	−6.25	**<0.001**
number of songs recorded	−0.10	0.05	−1.93	0.056

Exploration behaviour did not predict the number of syllables per song (table 3; figure 2). More aggressive individuals had fewer syllables per song (table 3; figure 3). Regardless of personality phenotype, fledglings had fewer syllables per song than adults, and males had fewer syllables per song than females (table 3). The number of syllables per song was not predicted by the number of songs recorded per individual (table 3). The random effect ‘Territory ID’ significantly accounted for variance in syllables per song (variance = 1.54, LRT = 7.92, d.f. = 1 and *p* = 0.005).

## 4. Discussion

Although personality traits and sexual signalling may interact in many ways [12,13], very few studies have explored these relationships, especially in species where both sexes sing. Here, we found different associations between song complexity and two personality traits (exploration and aggressiveness) in wild superb fairy-wrens. Specifically, individuals that were more exploratory (regardless of their sex or life stage) produced more element types per song. More aggressive fledglings, but not adults, also had more element types per song, and more aggressive individuals produced songs with fewer syllables regardless of their life stage. Independent of their personality phenotype, fledglings produced songs with fewer syllables than adults and males produced songs with fewer syllables than females. Our study is the first to highlight a link between personality and song complexity in birds and illustrates that both males and females can advertise their personalities when singing.

Two mechanisms could potentially explain the positive correlation we found between exploration phenotype and song complexity. First, personality may shape an individual’s song learning by influencing its attention to tutor social cues [33] and its speed of learning [30]. Fast explorers can learn faster than slow explorers (e.g. in cognitive tasks [30]), which could decrease song complexity when learning to sing if individuals do not allocate sufficient time to assimilate all relevant information (i.e. element types) within their environment. Our study, however, did not measure the accuracy or speed of vocal learning, so we cannot assess how well, or how quickly, fast- and slow-exploring birds copied their tutors’ songs. Second, an individual’s personality may influence its choice of song tutor [33]. Juvenile superb fairy-wrens learn song elements from both of their social parents [43], but it is unknown to what extent they also copy non-parental tutors, such as subordinate helpers or neighbours. It is plausible that more exploratory juveniles that stray farther from their parents are more likely to experience and memorize non-parental song elements, which may supplement their parental repertoires and thereby increase song complexity. A similar mechanism has been proposed in Australian zebra finches (*Taeniopygia castanotis*), where fast-exploring offspring were found to produce more non-paternal song syllables (i.e. syllables absent from their father’s repertoire) than slow-exploring offspring [33].

If an individual’s personality influences its song, then one function of birdsong may be to honestly signal the singer’s behavioural phenotype [67]. This is supported by our data, as more exploratory individuals sang more complex songs (which would be more desirable by mates; [68]) than less exploratory individuals. It is unknown, however, whether exploration behaviour predicts breeding success and mate choice in superb fairy-wrens, although comparable associations have been found in other songbird species [21,69,70]. Previous studies in superb fairy-wrens have shown that an individual’s exploration phenotype predicts its risk-taking behaviour during the breeding season, as well as its survival probability (i.e. more exploratory individuals were less likely to be alive 1 year later) [15,41]. Mate choice experiments would help in understanding the relationship between behavioural phenotypes and fitness. For example, in a study of zebra finches, females preferred to hear the songs of males that successfully solved a novel foraging task over the songs of males that did not, suggesting that song alone can potentially carry information about the singer’s behavioural type or cognitive ability [67].

We also found a relationship between aggressiveness and song complexity. First, we found that more aggressive individuals (regardless of their life stage or sex) produced songs with fewer syllables. This is not surprising considering that bolder and more aggressive birds are often less innovative than shyer/less aggressive birds [46,47], which could influence which and how many elements are incorporated into their songs and thus the number of song syllables. In Darwin’s small ground finches (*Geospiza fuliginosa*), aggressive males were more likely to produce a common rather than rare song syllable type, and aggressive males defended their territories more strongly against an intruder male with a common rather than a rare syllable type [71]. Alternatively, there may be a signal trade-off between song complexity (to attract mates) and song length (to deter intruders), which could affect the number of syllables used. Indeed, songs in birds are complex sexual signals that serve different functions [60], and individuals have been shown to signal aggressiveness in agonistic contexts by changing the structural characteristics of their songs (e.g. [72,73]) or their syllable/singing rate [72,74,75]. More aggressive individuals, by producing fewer songs to advertise their aggressiveness, may reduce the energy expenditure associated with longer songs [24,25] and preserve their energy for other aggressive behaviours. We also found that more aggressive fledglings, but not adults, had more element types per song, highlighting that the relationship between personality and song complexity can vary across life stages. More aggressive fledglings may experiment with a greater diversity of element types to assert dominance over other fledglings or prepare for the establishment of their own territory in their first year of life, leading to increasing song complexity. In contrast, adults have already settled in their territories and may not need to experiment as much. In this study, we measured individuals’ aggressiveness based on their time spent near the mirror during a mirror stimulation test. Although approaching one’s mirror image is usually interpreted as an aggressive response in fairy-wrens [15,76], such a response could also indicate sociability towards a perceived conspecific, particularly in fledglings that do not yet serve a defensive role in their natal territory. Hence, our results may instead indicate that more sociable fledglings produce more element types per song, perhaps because they spend more time close to conspecifics and learning their songs.

As expected, we found that recording effort (number of songs recorded per individual) influenced song characteristics, specifically the number of element types per song. Although superb fairy-wrens sing three distinct song types, no instance of the chatter song is identical in their syntax or elemental composition (Colombelli-Négrel, pers. obs.). Therefore, recording more songs per individual would increase not only the likelihood of capturing more rare element types but also enhance the opportunity to observe different syntaxes and consequently increase diversity in the number of element types used.

Consistent with previous studies [43,51,57,59], we found differences in song complexity between sexes. However, our results revealed less complexity in males’ songs, as they produced songs with fewer syllables than females (regardless of their personality phenotypes). A previous study showed that sexual selective pressures on songs can vary within and between fairy-wren species [77]. Specifically, the authors demonstrated that male and female songs were more similar when male survival rates were high and when parental care was more evenly shared [77]. Therefore, the differences in song complexity observed between this study and prior studies (including from the same population) may simply reflect differences in sex roles over time and space. Since superb fairy-wrens also do not differ in personality between sexes, and we found no significant interactions between sex and personality, it may be beneficial for future studies to investigate the relationship between personality, sex and song complexity in species that show sex-specific personality traits.

Taken together, these results demonstrate that learned aspects of sexual signalling are personality dependent, with potential fitness implications. We have shown that both males and females advertise their personalities when singing, highlighting the importance of studying species in which both sexes sing when disentangling the proximate and ultimate pressures that drive sexual signalling.

## Data Availability

Data provided as supplementary material [78].
